# Smart Pen Exposes Missed Basal Insulin Injections and Reveals the Impact on Glycemic Control in Adults With Type 1 Diabetes

**DOI:** 10.1177/19322968221104142

**Published:** 2022-07-01

**Authors:** Neda Rajamand Ekberg, Niels Væver Hartvig, Anne Kaas, Jonas Bech Møller, Peter Adolfsson

**Affiliations:** 1 Center for Diabetes, Academic Specialist Center, Stockholm, Sweden; 2 Department of Molecular Medicine and Surgery, Karolinska Institutet, Karolinska University Hospital, Stockholm, Sweden; 3 Novo Nordisk A/S, Bagsværd, Denmark; 4 Department of Pediatrics, The Hospital of Halland, Kungsbacka, Sweden; 5 Institute of Clinical Sciences, Sahlgrenska Academy at University of Gothenburg, Gothenburg, Sweden

**Keywords:** adherence, basal insulin, glycemic control, smart insulin pen, time in range, type 1 diabetes

## Abstract

**Background:**

Adherence to basal insulin injections and the effects of missed basal insulin injections in adults with type 1 diabetes (T1D) were investigated using data from continuous glucose monitoring (CGM) and smart insulin pen devices in a real-world study.

**Methods:**

This was a post hoc analysis of a prospective, real-world study conducted in Sweden. Adults with T1D who were using CGM received a smart insulin pen device (NovoPen 6) for insulin injections. Missed basal insulin doses (≥40 hours between doses) were evaluated over 14-day periods, and the probability of missing basal insulin doses was estimated. Associations between missed basal insulin doses and glycemic outcomes were also explored.

**Results:**

Thirty-two patients with 4410 acceptable CGM days (315 14-day periods) were included. The number of missed basal insulin doses ranged from 0 to 4 over 315 14-day periods. The estimated probability of missing at least one basal insulin dose over any given 14-day period was 22% (95% confidence interval: 10%-40%). Missed basal insulin doses were significantly associated with higher mean glycemic levels, higher glucose management indicator, and lower time in range (70-180 mg/dL [3.9-10.0 mmol/L]). Similar results were observed when adjusted for missed bolus insulin doses; age and sex had no statistically significant effect on any glycemic parameter.

**Conclusions:**

This is the first study, based on accurate real-world injection data, to demonstrate the challenge of adherence to basal insulin injections in patients with T1D, and document that just one missed basal injection per week can result in clinically significant changes in glycemic control.

## Introduction

Basal insulin injections are administered as part of a basal-bolus regimen and are central to glycemic management in patients with type 1 diabetes (T1D).^1^ For adults with new-onset T1D initiating insulin therapy, a basal-bolus regimen can be used to administer insulin, but other regimens, such as continuous subcutaneous insulin infusion (CSII), with and without long-acting insulin, can be used if these are more suitable for the patient.^2,3^ Intensive insulin therapy in patients with diabetes can delay the onset and progression of microvascular and macrovascular complications, as well as reduce morbidity and mortality.^4,5^ Despite these benefits of insulin therapy, there are several treatment barriers that can reduce insulin adherence, resulting in suboptimal glycemic control.^6^ As of 2020, in Sweden, only approximately 28% of adult patients with T1D receiving specialist diabetes care achieved a target glycated hemoglobin (HbA1c) level of <52 mmol/mol (6.9%), and approximately 26% of adults with T1D administered their insulin using CSII (with the remaining proportion of patients using basal-bolus regimens).^7^ Low insulin regimen adherence is associated with poor glycemic control, increased risk of diabetic ketoacidosis, and high rates of hospital admissions.^4,8-10^ Managing insulin treatment schedules around the variable demands of daily life can be challenging and, as a result, patients may skip or forget their insulin injections.^8,11^ In addition to impaired memory, other treatment barriers include high anxiety around self-injection, fear of hypoglycemia, and rising medication costs.^12^

Previous observational studies of basal insulin treatment patterns have been based on patient diaries and surveys^13,14^; such patient self-reported information regarding the actual insulin dose delivered and the precise timing of injections can be inaccurate or incomplete.^15^ Technological advancements in continuous glucose monitoring (CGM) and insulin delivery systems, and the emergence of digital technologies, such as connected insulin pen devices (or “smart insulin pens”),^16^ offer opportunities to optimize insulin management, reduce dosing errors, and improve treatment adherence.^17-19^

Given that previous studies investigating the impact of missing insulin injections on glycemic control have generally focused on bolus doses of insulin,^4,8^ there is a lack of data on the effect of missed basal insulin doses in T1D. The aim of this study was to investigate the effect of missed basal insulin injections on glycemic control and glucose variability using accurate data from a smart insulin pen device (NovoPen 6) collected in a real-world study.^17^ The adherence to basal insulin injections was investigated and the association of missed basal insulin doses with glycemic control in patients with T1D was explored, using data from both CGM and the smart insulin pen device.

## Methods

### Study Design

This was a post hoc analysis of a one-arm, prospective, proof-of-concept, real-world study conducted at 12 diabetes clinics across Sweden from May 2017 to April 2019, which analyzed missed basal insulin doses in patients with T1D.^17^ The study design has previously been described by Adolfsson et al.^17^ Participants with T1D who were already using CGM during the pre-baseline period were continuously enrolled into the study and were offered a smart insulin pen device (NovoPen 6) at the discretion of their treating physician. On accepting the pen, participants were asked to provide written informed consent allowing Glooko, Inc. (Mountain View, California) to collect the participant’s data and share them with Novo Nordisk for scientific purposes. Throughout the study duration, data were collected from patients using NovoPen 6 for insulin administration regarding the timing and dose of basal and/or bolus insulin injections. The complete data set, including CGM data, was downloaded from the Glooko database at the conclusion of the study.^17^ Swedish Ethics Committee approval (2019-01270) was obtained before any study-related activities commenced.

Participants were blinded to the injection data overview during the baseline period but continued to be able to see their CGM data and the dose of their previous injection displayed on the pen.^17^ At the first clinic visit, the initial set of injection data was downloaded for discussion between the participant and the health care practitioner (HCP).^17^ Thereafter, follow-up visits were scheduled according to usual clinical practice, and pen and CGM data were downloaded, discussed, and acted upon at each visit; CGM data were also uploaded between visits.^17^

### Post Hoc Analysis: Participants

This post hoc analysis included adults with T1D (≥18 years of age) who were using a smart pen for basal administration of insulin degludec with or without a smart pen for bolus insulin injections. In the present analysis, patients were included if they had at least one 14-day period with at least two basal injections and ≥70% CGM coverage. Given that only patients who were using a smart pen for basal insulin administration were included, fewer patients were analyzed in this analysis than in the primary analysis^17^ because most patients only used smart pens to administer their bolus insulin.

### Outcomes and Statistical Analyses

**Figure 1.**
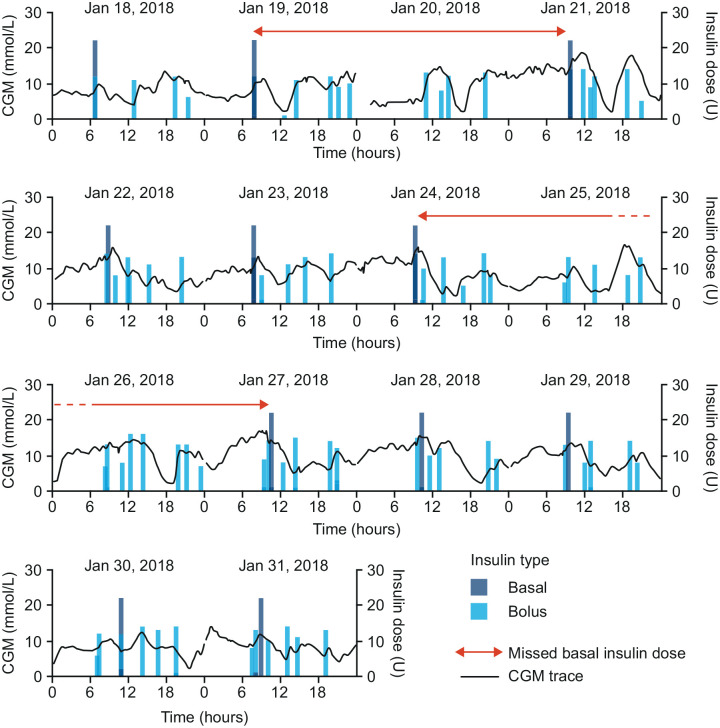
An example CGM trace illustrating how missed basal insulin doses were defined. This figure illustrates how a missed basal insulin was defined (ie, >40 hours between basal insulin doses). Short gaps in the CGM signal represent time points with missing data. Abbreviation: CGM, continuous glucose monitoring.

The numbers of missed basal insulin doses were assessed over 14-day periods (Figure 1). A missed basal insulin dose was defined as a period with ≥40 hours between two basal injections, in accordance with the definition for flexible dosing provided in the European summary of product characteristics for insulin degludec.^20^

To assess glycemic control, the following outcomes were aggregated over each 14-day period: time in range (TIR) (70-180 mg/dL [3.9-10.0 mmol/L]), time above range (TAR) level 1 (>180-250 mg/dL [>10.0-13.9 mmol/L]), TAR level 2 (>250 mg/dL [>13.9 mmol/L]), time below range (TBR) level 1 (54-<70 mg/dL [3.0-<3.9 mmol/L]), TBR level 2 (<54 mg/dL [<3.0 mmol/L]), mean glucose level, glucose management indicator (GMI),^21^ and % coefficient of variation (%CV).

As a covariate to use in the analysis, the number of missed bolus insulin doses was determined for patients who also had a connected bolus insulin pen. A missed bolus insulin dose was defined as a meal with no bolus injection within the window of 15 minutes before to one hour after the start of the meal. Meals were identified from the CGM signal using the clinically validated GRID algorithm.^22^ The algorithm identified meals at points when the CGM signal was ≥7.2 mmol/L (≥130 mg/dL) and the rate of change was ≥5.3 mmol/(L·hour) (≥95 mg/[dL·hour]) over two consecutive readings or ≥5.0 mmol/(L·hour) (≥90 mg/[dL·hour]) for two out of three consecutive readings. The CGM time series was resampled to 15-minute readings before the application of the algorithm.

Probabilities of missing basal insulin doses were estimated based on a cumulative linked mixed model, with patients as random effects. The model assigns separate probabilities to events of 0, 1, 2, or >2 missed basal insulin doses. Patients are included as random effects with a logit-link function to allow for differences between patients and the estimated probabilities can be interpreted as the probabilities for an average patient. The model was fitted using the software R and the function “clmm2” of the library “ordinal.” Approximate Wald-type confidence intervals were calculated, using the delta method to calculate standard errors of nonlinear combinations of parameters. To determine whether the probability was associated with age, TIR, or %CV, a stratified model was considered. For example, patients were stratified by age by dichotomizing age to either equal to or below the median value (31.1 years) or above the median value. For patients with a connected bolus insulin pen, the analysis was also stratified by “above” or “below” median number of missed bolus insulin doses. A model was fitted, in which the probabilities were allowed to depend on the two-level age factor, and a likelihood ratio test for homogeneity was conducted.

Associations between number of missed basal insulin doses and glycemic outcomes were estimated from linear mixed models with the patient identifier as a random intercept. Two models were used in this analysis: an unadjusted model and an adjusted model. In the unadjusted model, only the number of missed basal insulin injections over the 14-day period was included as a fixed effect as a continuous covariate. In this model, the effect of missed basal insulin doses was not adjusted for any potential confounders. In the adjusted model, the number of missed basal and bolus insulin doses and the age and sex of patients were included as covariates. Patients who did not use the connected pen for their bolus insulin injections were excluded from the second model, because the number of missed bolus insulin doses could not be calculated. The model was fitted using the software R and the library “lme4.”

## Results

### Patient Characteristics

Overall, 32 patients (mean age: 35.6 years) were included in this analysis, with 4410 acceptable CGM days (315 14-day periods). A summary of patient characteristics and key glycemic parameters is shown in Table 1. All patients included in this analysis received basal insulin degludec; 28 of the 32 patients also administered their bolus insulin injections using a smart insulin pen during the study.

**Table 1.** Patient Characteristics and Key Glycemic Parameters at Baseline.

Characteristics/parameters	N = 32
Age (average in study period), years^a,b^	35.6/31.1 (15.9) [18.1-72.8]
Sex, n (%)	Male: 17 (53.1); female: 15 (46.9)
14-day periods, N (N/patient)	315 (9.8)
Number of days, N (N/patient)	4410 (137.8)
Period	May 1, 2017 to April 6, 2019
Bolus pen, n (%)	Yes: 28 (87.5); no: 4 (12.5)
Bolus insulin, n (%)	IAsp: 26 (92.9); faster aspart: 1 (3.6); IAsp/faster aspart: 1 (3.6)
Basal pen, n (%)	Yes: 32 (100)
Basal insulin, n (%)	Insulin degludec: 32 (100)
TIR, %^c^	39.4 (13.9)
TBR L1, %^c^	3.2 (2.3)
TBR L2, %^c^	2.8 (3.5)
TAR L1, %^c^	24.8 (6.5)
TAR L2, %^c^	29.8 (14.4)
Mean glucose, mmol/L^c^	11.3 (2.1)
%CV glucose, %^c^	42.6 (8.2)
GMI,^21^ %^c^	8.2 (0.9)

Abbreviations: %CV, % coefficient of variation; GMI, glucose management indicator; IAsp, insulin aspart; SD, standard deviation; TAR, time above range (level 1, >180-250 mg/dL [>10.0-13.9 mmol/L]; level 2, >250 mg/dL [>13.9 mmol/L]); TBR, time below range (level 1, 54-<70 mg/dL [3.0-<3.9 mmol/L]; level 2, <54 mg/dL [<3.0 mmol/L]); TIR, time in range (70-180 mg/dL [3.9-10.0 mmol/L]).

a Age calculated at start of each 14-day period.

b Mean/median (SD) [range].

c Mean (SD).

### Estimated Probability of Missing Basal Insulin Doses

**Figure 2.**
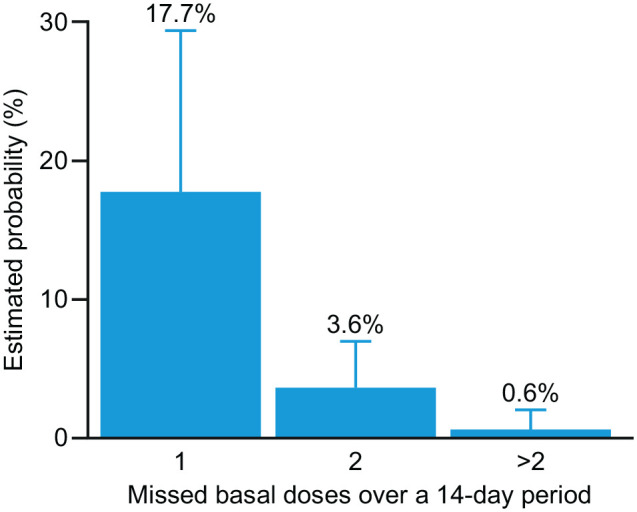
Estimated probability of missed basal insulin injections during 14-day periods. The probability that an average patient missed at least one basal insulin dose during a 14-day period was estimated to be 22% (95% CI: 10%-40%). Error bars represent 95% CI values. Abbreviation: CI, confidence interval.

The number of missed basal insulin doses varied between zero and four over the 315 14-day periods. The estimated probabilities of missing 1, 2, or >2 basal insulin doses during a 14-day period are shown in Figure 2 and Table 2. The estimated probability that an average patient missed at least one basal insulin dose was 22% (95% confidence interval: 10%-40%).

**Table 2.** Estimated Probability of Missing Basal Insulin Doses During a 14-Day Period; N = 32.

Model and subgroup	Number of missed basal insulin doses over a 14-day period				Test for homogeneity^a^
	*≥1*	*1*	*2*	*>2*	
Unstratified model	**21.9 (10.5, 89.5)**	17.7 (6.1, 29.3)	3.6 (0.3, 6.9)	0.6 (0.2, 2.0)	NA
Stratified model	–	–	–	–	–
High %CV (>42%)	**46.6 (25.3, 74.7)**	34.1 (19.5, 48.7)	10.4 (1.4, 19.4)	2.1 (0.7, 6.7)	.007
Low %CV (≤42%)	**7.3 (2.3, 97.7)**	6.4 (0.0, 13.6)	0.9 (0.0, 2.4)	0.0 (0.0, 100.0)	
High TIR (>39.2%)	**26.4 (10.7, 89.3)**	22.3 (5.0, 39.6)	3.5 (0.0, 7.6)	0.5 (0.1, 2.5)	.313
Low TIR (≤39.2%)	**17.3 (5.4, 94.6)**	12.5 (0.0, 25.5)	4.1 (0.0, 9.5)	0.8 (0.1, 4.0)	
High missed bolus insulin (>14.1 doses)	**30.4 (12.2, 87.8)**	21.6 (5.7, 37.5)	7.3 (0.0, 15.3)	1.6 (0.4, 6.5)	.188
Low missed bolus insulin (≤14.1 doses)	**18.6 (6.4, 93.6)**	16.2 (0.6, 31.8)	2.2 (0.0, 5.1)	0.2 (0.0, 1.9)	
Age >31.1 years	**18.0 (6.8, 93.2)**	15.0 (1.7, 28.3)	2.7 (0.0, 5.8)	0.3 (0.1, 1.8)	.647
Age ≤31.1 years	**28.1 (10.8, 89.2)**	21.2 (4.2, 38.1)	5.7 (0.0, 12.4)	1.2 (0.3, 5.3)	

This table contains the estimated probability with 95% confidence intervals of missing different numbers of basal insulin doses over a 14-day period. The gray column represents the overall probability of missing any number of basal injections, and columns 1, 2, and >2 contain the estimated probabilities of missing a specific number. The estimates are based on a cumulative linked mixed model with patient as random effect. Confidence intervals for linear combination of parameters are based on the delta method. In the stratified models, patients were stratified into two groups of equal size, with different probabilities for each group. N = 28 in the analysis stratified by missed bolus dose.

Abbreviations: %CV, % coefficient of variation; NA, not applicable; TIR, time in range (70-180 mg/dL [3.9-10.0 mmol/L]).

a Homogeneity test was an analysis of variance test comparing the stratified model (stratified by %CV, missed bolus insulin dose, age, and TIR) and unstratified model.

The estimated probabilities of missing a basal insulin dose, stratified by %CV, TIR, missed bolus insulin dose, and age, are shown in Table 2. Among patients with a high %CV, there was a higher probability of at least one basal insulin dose being missed than among patients with a low %CV (*P* = .007). There was a non-statistically significant increase in the probability of missing at least one basal insulin dose in patients who also missed many bolus insulin doses compared with those who missed fewer bolus insulin doses. No statistically significant differences were observed for the probability of missing basal insulin injections in patients with high versus low TIR, or for younger versus older patients.

### Associations Between Missed Basal Insulin Doses and Glycemic Parameters

The estimated associations between missed basal insulin doses and glycemic parameters, adjusted for age, sex, and missed bolus doses, are presented in Table 3. The associations in the unadjusted model are presented in Supplemental Table S1.

**Table 3.** Estimated Change in Glycemic Parameters Per Missed Basal Insulin Dose and Per Missed Bolus Insulin Dose, Respectively, Based on the Adjusted Linear Mixed Model; N = 28.

Glycemic parameters	Estimated mean change per missed basal insulin injection (95% CI)	*P*	Estimated mean change per missed bolus insulin injection (95% CI)	*P*
TIR, %	−2.63 (−4.41, −0.71)	.005	−0.25 (−0.44, −0.07)	.008
TAR L1, %	0.34 (−0.71, 1.34)	.520	−0.03 (−0.14, 0.07)	.525
TAR L2, %	2.91 (0.99, 4.73)	.002	0.26 (0.07, 0.45)	.008
TBR L1, %	−0.28 (−0.65, 0.11)	.154	0.00 (−0.03, 0.04)	.808
TBR L2, %	−0.29 (−0.78, 0.22)	.256	0.02 (−0.04, 0.06)	.556
Mean glucose, mmol/L	0.44 (0.19, 0.69)	<.001	0.02 (−0.00, 0.05)	.085
%CV, %	−0.09 (−1.08, 0.95)	.855	0.19 (0.09, 0.29)	<.001
GMI, %	0.19 (0.08, 0.30)	<.001	0.01 (−0.00, 0.02)	.085

The estimates are based on a linear mixed model with number of missed bolus insulin doses, number of missed basal insulin doses, age, and sex as fixed effects and patients as random effects.

Abbreviations: CI, confidence interval; %CV, % coefficient of variation; GMI, glucose management indicator; TAR, time above range (level 1, >180-250 mg/dL [>10.0-13.9 mmol/L]; level 2, >250 mg/dL [>13.9 mmol/L]); TBR, time below range (level 1, 54-<70 mg/dL [3.0-<3.9 mmol/L]; level 2, <54 mg/dL [<3.0 mmol/L]); TIR, time in range (70-180 mg/dL [3.9-10.0 mmol/L]).

Each missed basal insulin dose was associated with an increase in the mean glycemic level (*P* < .001), a reduced TIR (*P* = .005), an increased TAR L2 (*P* = .002), and higher GMI (*P* < .001) (Table 3). Each missed basal dose corresponds to a reduction of TIR of −2.6% and an increase in mean glucose of 0.44 mmol/L. Thus, two missed basal insulin doses correspond to a change in TIR of −5.3%; a difference of 5% is considered clinically relevant.^23^ Missed basal insulin doses did not lead to a higher %CV (*P* = .855). Thus, the association seen earlier between missed basal insulin doses and patients with high %CV is not causal but is more likely a result of other factors affecting both basal insulin doses and %CV, such as patients who also frequently miss bolus doses. In contrast to this, %CV was significantly associated with missed bolus doses (*P* < .001) and, as such, missed bolus doses were also associated with TIR (*P* = .008) and TAR L2 (*P* = .008).

Age and sex did not have a statistically significant effect on any of the glycemic parameters (Supplemental Table S2).

The results of the unadjusted model (Supplemental Table S1) were consistent with the results of the adjusted model (Table 3).

## Discussion

To the best of our knowledge, this is the first evidence, based on accurate real-world insulin dose data, to demonstrate that adherence to basal insulin injections can be a challenge for patients with T1D. The present study was the first to investigate the effects of missed basal insulin injections using accurate injection data from a smart insulin pen; previous studies using data from smart insulin pens have primarily focused on missed bolus insulin doses.^4,8^

Herein, the estimated probability that an average patient missed at least one basal insulin dose in a 14-day period was 22%. The probability of missing a basal insulin dose was associated with glycemic variability; a higher probability of missing a basal insulin dose was observed for patients with high %CV than for patients with low %CV. This is likely not a causal effect since the analysis of the association between the number of missed basal insulin doses and glycemic parameters indicated that, for each patient, a change in the number of missed basal insulin doses does not increase the %CV. The association is therefore likely explained by a confounder that affects both the %CV and the overall likelihood of patients missing basal insulin doses, for instance, as the level of engagement in treatment or overall life circumstances. There was a trend, although not statistically significant, for patients who missed a bolus insulin dose to have a higher probability of missing a basal insulin dose than those who did not miss their bolus insulin dose. TIR and age were not associated with the probability of missing a basal insulin dose, indicating that younger patients did not forget more basal insulin doses than older patients.

The mixed model analysis of the glycemic parameters illustrated the within-patient effect of variation in the number of missed basal insulin doses. Notice that the glycemic parameters are inter-related, reflecting different, but related, aspects of the underlying glucose profile. We found that for each patient, an increase in the missed basal insulin doses were associated with increases in the mean glycemic level and decreases in TIR, but there was no direct statistically significant effect on glycemic variability (Table 3), despite the fact that the %CV is a function of the mean glucose. One missed basal insulin dose per week (or two missed basal insulin doses during a two-week period) was sufficient to correspond to a clinically relevant (a difference of ≥5%) change in TIR (−5.3%).^23^ In contrast, missed bolus insulin doses were associated with changes in glycemic variability and, as such, a reduced TIR. This is consistent with other studies of patients with T1D that have demonstrated that missed bolus insulin doses correlate with higher HbA1c levels.^24,25^ We did not find an association between age and sex and the key glycemic parameters. Collectively, these data demonstrate that patients with high glycemic variability tended to forget more basal insulin doses, but that high glycemic variability was not a direct result of the missed basal insulin doses.

The observed associations between missed basal insulin doses and reduced glycemic control highlight a need for tools, such as smart insulin pens and other technologies, that provide precise injection data and empower patients and health care practitioners (HCPs) to improve the treatment of diabetes.^13,15^

The patients in this study were using a smart insulin pen (NovoPen 6) in conjunction with CGM, but still experienced missed basal insulin doses; therefore, the addition of a smart insulin pen alone may not be sufficient to ensure 100% adherence to diabetes therapies among patients with T1D. However, the use of smart insulin pen data may result in better treatment outcomes by enabling patients and HCPs to identify problems, such as missed basal insulin doses. Additionally, as demonstrated in a previous analysis using data from the same study, using a smart insulin pen (such as NovoPen 6) in conjunction with CGM may help to reduce the number of missed bolus insulin doses, improve glycemic control, decrease glucose variability, and increase treatment concordance among patients with T1D.^17^ Results from a three-year follow-up study of adults with T1D showed comparable improvements from baseline in glycemic levels when CGM was used with basal-bolus treatment and with CSII, highlighting the importance of CGM in both T1D treatment regimens.^26^ With added functions, such as a direct and downloadable memory, the use of smart pens may further improve efficacy of CGM in patients with T1D. Moreover, because NovoPen 6 is provided with an open application programming interface, this smart insulin pen can work in conjunction with smart phone apps with basal injection reminders or with different CGM devices, providing flexible options and additional benefits for patients and HCPs.

A key strength of this analysis was that the use of smart insulin pens enabled the collection of accurate injection timing data under real-world conditions, and, to our knowledge, this was the first study to evaluate the association between clinical outcomes and basal insulin dose patterns. However, there are limitations, such as the small study population and limited background information on the included patients, which should be considered when interpreting these results. Although, on average, more than three months of data were collected per patient, these data were grouped into 14-day intervals for simplicity and for consistency with common recommendations for summarizing the CGM data.^27^ This was an intentional choice to have a transparent definition of missed basal insulin doses, which span periods longer than a day, and to provide a relatively simple analysis framework, avoiding some of the issues of temporal correlation that arise when analyzing data on a finer time scale. Consequently, the model of exposure and outcome is coarse, and we cannot include temporal covariates, such as time since last missed basal insulin dose in this analysis. Also, since an HCP visit may fall before, during, or after a 14-day period, a detailed study of the effect of the HCP visit with the smart pen and changes over time in basal insulin adherence is not easily included in this framework. These are questions for future studies, where data collection over longer time intervals may be relevant and where also the analysis of injection behavior among patients with type 2 diabetes in future would be valuable.

## Conclusions

This is the first study to demonstrate, based on real-world injection data collected from smart insulin pens, that adherence to basal insulin injections can be a real challenge for patients with T1D and that missed basal insulin injections have a considerable impact on glycemic control. Furthermore, using smart insulin pen devices in conjunction with CGM could enable the timely detection of missed basal insulin injections, improving the quality of discussions between HCPs and patients, thereby increasing patient awareness, empowering patients to improve their diabetes management, and providing ongoing diabetes management education and support.

## Supplemental Material

sj-docx-1-dst-10.1177_19322968221104142 – Supplemental material for Smart Pen Exposes Missed Basal Insulin Injections and Reveals the Impact on Glycemic Control in Adults With Type 1 Diabetes Supplemental material, sj-docx-1-dst-10.1177_19322968221104142 for Smart Pen Exposes Missed Basal Insulin Injections and Reveals the Impact on Glycemic Control in Adults With Type 1 Diabetes by Neda Rajamand Ekberg, Niels Væver Hartvig, Anne Kaas, Jonas Bech Møller and Peter Adolfsson in Journal of Diabetes Science and Technology
