# Supplementary material for: Smart Pen Exposes Missed Basal Insulin Injections and Reveals the Impact on Glycemic Control in Adults With Type 1 Diabetes
Source: J Diabetes Sci Technol. 2022 Jul 1;18(1):66–73. doi: 10.1177/19322968221104142 (PMC10899845; doi:10.1177/19322968221104142)
Supplement: sj-docx-1-dst-10.1177_19322968221104142 – Supplemental material for Smart Pen Exposes Missed Basal Insulin Injections and Reveals the Impact on Glycemic Control in Adults With Type 1 Diabetes [file sj-docx-1-dst-10.1177_19322968221104142.docx]

**Supplementary Table S1.** Estimated Change in Glycemic Parameters Per Missed Basal Insulin Dose Based on the Unadjusted Linear Mixed Model; *N* = 32

| Glycemic parameters | Estimated change per missed basal insulin injection, mean (95% CI) | *P* |
| --- | --- | --- |
| TIR, % | −2.61 (−4.11, −1.11) | <.001 |
| TAR level 1, % | 0.36 (−0.46, 1.17) | .385 |
| TAR level 2, % | 2.89 (1.42, 4.34) | <.001 |
| TBR level 1, % | −0.23 (−0.56, 0.10) | .172 |
| TBR level 2, % | −0.35 (−0.80, 0.11) | .125 |
| Glucose concentration, mmol/L | 0.44 (0.24, 0.64) | <.001 |
| %CV, % | −0.05 (−0.93, 0.86) | .913 |
| GMI, % | 0.19 (0.10, 0.28) | <.001 |

The estimates are based on a linear mixed model with number of missed basal insulin doses as fixed effect and patient as random effect.

%CV, % coefficient of variation; CI, confidence interval; GMI, glucose management indicator; TAR, time above range (level 1, >180-250 mg/dL [>10.0-13.9 mmol/L]; level 2, >250 mg/dL [>13.9 mmol/L]); TBR, time below range (level 1, 54-<70 mg/dL [3.0-<3.9 mmol/L]; level 2, <54 mg/dL [<3.0 mmol/L]); TIR, time in range (70-180 mg/dL [3.9-10.0 mmol/L]).

**Supplementary Table S2.** Estimated Relationship Between Glycemic Parameters and Age and Gender, Respectively, Based on the Adjusted Linear Mixed Model; *N* = 28

| Glycemic parameters | Estimated change per 10 years of age, mean (95% CI) | *P* | Estimated difference men versus women, mean (95% CI) | *P* |
| --- | --- | --- | --- | --- |
| TIR, % | 0.99 (−1.50, 3.51) | .456 | −0.34 (−8.30, 7.62) | .936 |
| TAR L1, % | 1.12 (−0.06, 2.30) | .082 | −0.59 (−4.34, 3.14) | .765 |
| TAR L2, % | −2.07 (−5.16, 0.99) | .209 | 1.40 (−8.42, 11.22) | .788 |
| TBR L1, % | 0.00 (−0.59, 0.58) | .990 | −0.05 (−1.91, 1.82) | .962 |
| TBR L2, % | −0.09 (−1.00, 0.82) | .849 | −0.26 (−3.16, 2.65) | .867 |
| Mean glucose, mmol/L | −0.28 (−0.73, 0.17) | .245 | 0.17 (−1.26, 1.60) | .821 |
| %CV, % | −0.73 (−2.54, 1.09) | .449 | 1.14 (−4.67, 6.96) | .711 |
| GMI, % | −0.12 (−0.31, 0.07) | .245 | 0.07 (−0.54, 0.69) | .821 |

The estimates are based on a linear mixed model with number of missed bolus insulin doses, age, and sex as fixed effects and patient as random effect.

%CV, % coefficient of variation; CI, confidence interval; GMI, glucose management indicator; TAR, time above range (level 1, >180-250 mg/dL [>10.0-13.9 mmol/L]; level 2, >250 mg/dL [>13.9 mmol/L]); TBR, time below range (level 1, 54-<70 mg/dL [3.0-<3.9 mmol/L]; level 2, <54 mg/dL [<3.0 mmol/L]); TIR, time in range (70-180 mg/dL [3.9-10.0 mmol/L]).
